# Effectiveness and Safety of Bedaquiline-Containing Modified Shorter Regimens for Multidrug- or Rifampicin-Resistant Tuberculosis: A Single-Arm Meta-Analysis

**DOI:** 10.3390/pathogens15020130

**Published:** 2026-01-25

**Authors:** Yihui Zhou, Hongxia Niu

**Affiliations:** 1 The Fourth School of Clinical Medicine, Zhejiang Chinese Medical University, Hangzhou 310053, China; zyh7402025@outlook.com; 2 School of Basic Medical Science, Zhejiang Chinese Medical University, Hangzhou 310053, China

**Keywords:** multidrug-resistant tuberculosis, rifampicin-resistant tuberculosis, bedaquiline, meta-analysis

## Abstract

Tuberculosis (TB) remains a global public health emergency, with multidrug-resistant TB (MDR-TB) and rifampicin-resistant TB (RR-TB) posing critical challenges. Conventional longer regimens are characterized by suboptimal effectiveness, high toxicity, and poor tolerability. Consequently, there is an urgent demand for more effective, safer, shorter regimens with enhanced tolerability to replace traditional treatments. The present study aimed to systematically assess the effectiveness and safety of bedaquiline-containing modified shorter regimens (adaptations of the WHO-recommended 9–12-month bedaquiline-containing shorter regimen, with ethionamide, ethambutol, isoniazid, and pyrazinamide partially or fully substituted by linezolid, cycloserine/terizidone, and/or delamanid) for MDR/RR-TB. Databases (PubMed, Cochrane Library, Embase, and Web of Science) were searched up to 17 December 2025. Data on treatment success, adverse events, and patient characteristics were extracted. Heterogeneity was assessed using Cochrane Q test and I^2^ statistic. Eleven studies involving 8166 patients were included. The pooled treatment success rate was 78.5% (95% CI: 0.69~0.87, I^2^: 98.45%; *p* = 0.00). The incidence of serious adverse events was 10.0%. Bedaquiline-containing modified shorter regimens may offer a potentially viable treatment option for MDR/RR-TB patients, giving an option for patients who are ineligible for standardized regimens. In order to verify these findings, further large-scale trials are required.

## 1. Introduction

Tuberculosis (TB) remains a global public health emergency, with 10 million new cases annually, making it the leading cause of adult infectious disease mortality worldwide [1]. Drug resistance in tuberculosis has emerged as a major worldwide concern in recent years. According to the World Health Organization (WHO) Operational Handbook, TB disease brought on by a strain of the M. TB complex that is resistant to rifampicin is known as rifampicin-resistant TB (RR-TB). Multidrug-resistant TB (MDR-TB) occurs when these strains are also resistant to both isoniazid and rifampicin [2]. In 2023, the global number of MDR/RR-TB cases reached 175,923, with a treatment success rate of just 68%, significantly lower than the 88% success rate for drug-sensitive TB. Additionally, the WHO’s End TB Strategy aims for a treatment success rate above 90%, a target that remains unmet. These findings highlight the ongoing public health challenge posed by the global TB epidemic [3].

It is important to point out that the WHO Consolidated Guidelines recommend that effective treatment for drug-resistant TB requires the use of multiple medications over an extended period, often lasting 20 months or more [4]. For most RR/MDR-TB patients, treatment regimens typically combine various second-line drugs for up to 20 months. However, these regimens are often characterized by low effectiveness, high toxicity, prolonged treatment durations, and significant treatment challenges [5,6]. Therefore, there is an urgent need for more effective, safer, and shorter regimens with better tolerability to replace conventional treatments.

In contrast, shorter treatment regimens, lasting less than 12 months, appear to offer greater benefits. One study found that shorter regimens are noninferior to longer ones in terms of effectiveness and similar in safety [7]. Additionally, shorter regimens are associated with lower mortality and loss to follow-up rates [8,9] and they help reduce the economic burden on patients [10,11], better meeting the treatment needs of RR/MDR-TB patients. It is noteworthy that the WHO has recommended a shorter bedaquiline-containing regimen for eligible patients with RR/MDR-TB. This WHO-recommended bedaquiline-containing shorter regimen, which spans a duration of 9–12 months, incorporates levofloxacin or moxifloxacin, ethionamide, ethambutol, isoniazid, pyrazinamide, and clofazimine. It is intended for patients who do not exhibit resistance to fluoroquinolones [4].

But In clinical practice, standardized shorter regimens face limitations due to restricted medication options, strict application criteria, and fixed drug compositions, which reduce their applicability to diverse patient needs [12]. As a result, these regimens may not meet the treatment requirements of patients with varying conditions. To address these limitations, many recent studies have explored modified shorter regimens [13,14,15,16,17,18], which are defined as adapted versions of the standardized WHO-recommended bedaquiline-containing shorter regimen [14]. These adaptations involve replacing one or more component drugs, such as ethionamide, ethambutol, isoniazid, and pyrazinamide, with alternative agents like linezolid, cycloserine/terizidone, or delamanid, to better address local patterns of drug resistance or patient-level clinical needs.

Research shows that modified shorter treatment regimens provide options for patients who are ineligible for standardized regimens [19]. These regimens are generally well-accepted, with high adherence rates and improved patient satisfaction [20]. However, there is a need for more clinical, individualized alternatives that use drugs with fewer side effects and are tailored to patient needs. Adjustable modified regimens may offer better treatment options in the future [21]. Moreover, the WHO Consolidated Guidelines recommend all-oral, shorter regimens based on bedaquiline, pretomanid, and linezolid for MDR/RR-TB. These regimens, such as BPaL and BPaLM, have improved outcomes and reduced treatment duration compared with longer regimens [4,22].These evolving standards provide a contemporary context for understanding modified, shorter regimens, which similarly aim to optimize drug combinations and duration based on patient-level resistance profiles and tolerability. With the discovery of more potent drugs for TB treatment, such as bedaquiline, pretomanid, and delamanid, as well as repurposed drugs that have new therapeutic usages of already approved and established drugs (such as linezolid and clofazimine), the combination of these drugs has enabled a shift to shorter, more comprehensive, adjustable regimens [23].

In 2019, the WHO evaluated a modified 9-month treatment regimen based on the bedaquiline-containing regimen. Originally, this regimen included ethionamide, ethambutol, isoniazid, and pyrazinamide, but these drugs were replaced with linezolid, cycloserine, and delamanid (either individually or in combination), creating a tailored regimen for the local population and offering an alternative shorter treatment option [13]. Additionally, a phase III clinical trial explored shorter, safer, and more effective regimens using new drugs (bedaquiline and delamanid) and repurposed drugs (clofazimine and linezolid), showing favorable results and supporting simplified drug combinations. These regimens provide alternatives to treatments with severe side effects, interactions, contraindications, resistance, and unavailability, all based on individual patient characteristics [24].

The WHO currently supports further exploration of modified shorter regimens. However, there is limited evidence regarding their effectiveness and safety, and no studies have systematically evaluated these innovative regimens. Therefore, this study aims to evaluate the effectiveness and safety of modified shorter regimens. The modified shorter regimen in this study is based on the WHO-recommended bedaquiline-containing regimen, which includes levofloxacin or moxifloxacin, ethionamide, ethambutol, isoniazid, pyrazinamide, and clofazimine. In the adapted version, ethionamide, ethambutol, isoniazid, and pyrazinamide are replaced, either fully or partially, with linezolid, cycloserine/terizidone, and delamanid (individually or in combination).

## 2. Materials and Methods

### 2.1. Protocol and Registration

We prospectively registered this single-arm meta-analysis on PROSPERO (CRD420251000709), and this study was conducted using the Preferred Reporting Items for Systematic Reviews and Meta-Analyses (PRISMA) guidelines [25] (Supplementary File S1).

### 2.2. Search Strategy

Databases including PubMed, Embase, Web of Science, and Cochrane Library were searched, with the last search carried out on 17 December 2025. The search strategy involved a combination of subject and free words, with the free words being qualified by title or abstract.

Search terms related to the population partially included “TB, multidrug resistant [MeSH Terms,” “multidrug resistant TB,” “TB mdr,” “rifampicin-resistant TB,” “RR-TB,” and “RR-TB.”

Search terms for intervention partially included “short treatment regimen,” “shortened treatment regimens,” “modified short treatment regimen,” and “modified short treatment regimen.”

Search terms related to study type partially included “randomized controlled trial,” “randomized,” “Case-Control Studies [Mesh,” “Cohort Studies [Mesh,” and “Cohort Study.”

The complete search strategy is listed in the Supplementary Materials (Supplementary File S2).

### 2.3. Study Selection

The records of the searches from each database were summarized, and the literature was managed using EndNote X7 software. Two researchers independently screened the records that met the search strategy by title or abstract, then excluded any studies that did not fulfill the inclusion criteria or were duplicates from the initial screening. If browsing the titles and abstracts is not possible, the full text of the studies will be assessed, and non-compliant studies will be excluded in the secondary selection. Discussions and consultations will be held with a third researcher where any disagreements and discrepancies are involved.

All studies on the reported effectiveness and safety of modified shorter regimens containing bedaquiline for treating MDR/RR-TB patients were eligible for inclusion. Meanwhile, only randomized controlled trials (RCTs), cohort studies, or case–control studies published in English were included. The modified shorter regimen in this study is based on the WHO-recommended bedaquiline-containing regimen, which includes levofloxacin or moxifloxacin, ethionamide, ethambutol, isoniazid, pyrazinamide, and clofazimine. In the adapted version, ethionamide, ethambutol, isoniazid, and pyrazinamide are replaced, either fully or partially, with linezolid, cycloserine/terizidone, and delamanid (individually or in combination). The following exclusion criteria were used: (1) Literature was a review, case report, meta-analysis, editorial, letter, comment, protocol, or conference abstract; (2) The study population was non-human; (3) Drug sensitivity testing was not performed; (4) The patients were resistant to fluoroquinolones; (5) The treatment duration was longer than 12 months. (6) low quality assessment.

Treatment outcomes were recorded following the official definitions according to the guidelines: Treatment success is the sum of the number of patients who were cured and of those who completed treatment; death is defined as death from any cause that occurs during treatment; treatment failure, A TB patient whose sputum smear or culture is positive at month 5 or later during treatment [26]. Serious adverse events were defined as any adverse event that resulted in either death, permanent/significant disability or incapacity, hospitalization or prolongation of hospitalization to manage the adverse event or was categorized as life-threatening.

### 2.4. Quality Assessment

Different types of studies were assessed using different quality assessment tools. The Cochrane Risk of Bias Assessment Tool was used to evaluate the quality of RCTs, while the Newcastle–Ottawa Scale (NOS) was used to evaluate the quality of observational studies. RCTs were evaluated according to the six domains, including selection bias, performance bias, detection bias, attrition bias, reporting bias, and other bias. Each domain was graded as low, unclear, or high risk based on specific criteria.

The NOS scores three domains of population selection, comparability, and outcomes, with scores of up to 4, 2, and 3, for a total of 9. Selection focuses on the representativeness of study populations, reliability of exposure/case definition, and temporal sequence between exposure and outcome. Comparability focuses on the control of confounding factors to reduce internal bias, the only domain allowing cumulative scoring. Outcomes focuses on the reliability of outcome measurement and follow-up completeness for cohort studies. For case–control studies, it focuses on the consistency of exposure measurement. Studies with higher scores indicated better quality. Scores less than 4, 4–6, and higher than 6 are, respectively, considered low, moderate, and high quality. Two researchers conducted a quality assessment and resolved discrepancies through discussion. Studies with a NOS score of less than 4 and RCTs with 2 or more domains at high risk of bias were excluded.

### 2.5. Data Extraction

The required Information and data were extracted by two researchers into a preformed spreadsheet independently, and a discussion was held on any discrepancies that had been identified in the extracted data. We extracted data including first author, year of publication, country, study type, number of participants, patient characteristics (age, gender, human immunodeficiency virus [HIV status), drug resistance profile, treatment regimen, duration of treatment, outcomes, and adverse events.

### 2.6. Statistical Analysis

Statistical analyses were performed using Stata 14.0 software (Stata Corp, College Station, TX, USA). Since the outcomes were single-arm proportions, the data did not follow a normal distribution. To address variance instability and include studies with zero events, we applied the Freeman-Tukey double arcsine transformation to the raw proportions using the formula.

$$t=arcsin(\sqrt{n/(N+1)})+arcsin(\sqrt{\left(n+1)/(N+1\right)})$$

This transformation stabilizes the variance (Var=1/(N+0.5)) and inherently incorporates continuity correction. The meta-analysis was then conducted using the generic inverse variance method on the transformed values. Given the significant heterogeneity observed among the included studies ($I^{2}>50\%$), a random-effects model (DerSimonian-Laird method) was employed to pool the effect sizes. The pooled transformed proportions and their 95% confidence intervals (CIs) were back-transformed to the original scale for interpretation. Heterogeneity was assessed using the $I^{2}$ statistic and Cochran’s Q test. If significant heterogeneity was noted in the meta-analysis, subgroup analyses were conducted to explore potential sources of variability. Sensitivity analyses were also conducted.

### 2.7. Quality of Evidence

Evidence certainty was evaluated using the GRADE (Grading of Recommendations Assessment, Development, and Evaluation) approach. Each outcome’s evidence quality was assessed across five domains: risk of bias, inconsistency, indirectness, imprecision, and publication bias. Following the GRADE guidelines, overall evidence certainty was rated as high, moderate, low, or very low.

## 3. Results

### 3.1. Study Selection

**Figure 1:**
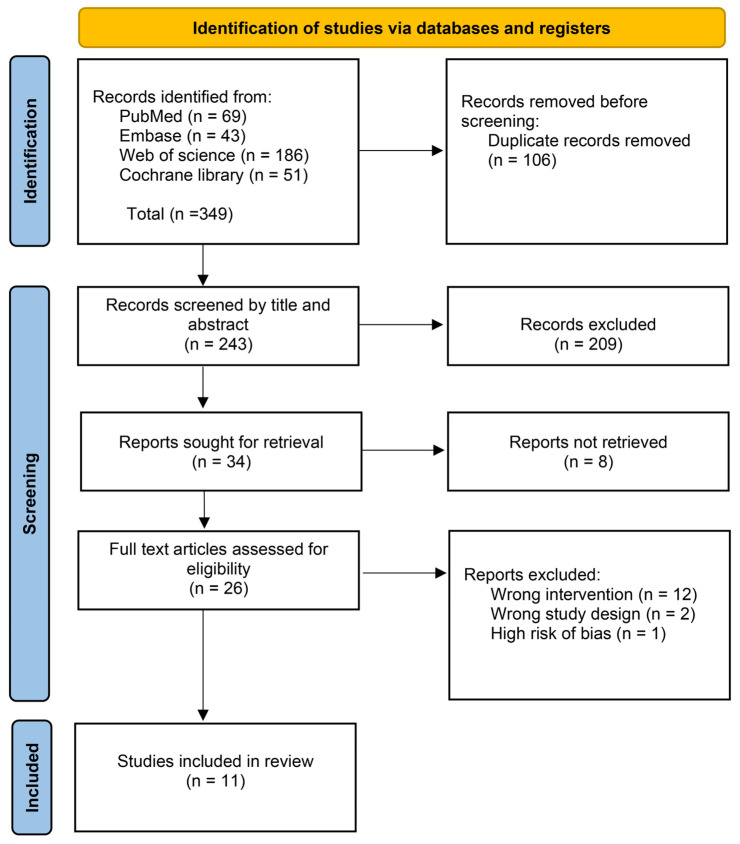
PRISMA flowchart of study inclusion.

We searched four databases, including PubMed, Embase, Web of Science, and Cochrane Library, and obtained 349 relevant studies. 338 studies were excluded for various reasons. Following the exclusion of 106 duplicates and the browsing of titles and abstracts of other identified studies, 34 were retained. After rescreening the full text of the studies, 12 were excluded due to wrong intervention, 2 were excluded due to wrong study design, 1 was excluded due to high risk of bias [27], and the exclusion of 8 items was due to unavailability of the full text. Ultimately, this meta-analysis included 11 studies that met the selective criteria [13,15,16,17,24,28,29,30,31,32,33] (Figure 1) and 8166 patients.

### 3.2. Study Characteristics

The following section presents the baseline characteristics of the included studies (Table 1). The included studies were mostly observational studies, including 6 prospective cohort studies, 2 retrospective cohort study, 1 retrospective and prospective comparative cohort study, and only 2 RCTs were included. The year of publication ranged from 2022 to 2025. Regarding the treatment regimen, all the studies included were all-oral regimens, 5 were modified shorter regimens with complete substitution, and 6 were shorter regimens with partial substitution. The total number of study participants included in the 11 studies was 8166, with a male-to-female ratio that exceeded 50%. HIV status was reported in 8 of the studies, with 40.3% of the patients being infected with HIV (*n* = 3296/8166).

### 3.3. Quality Assessment

**Figure 2:**
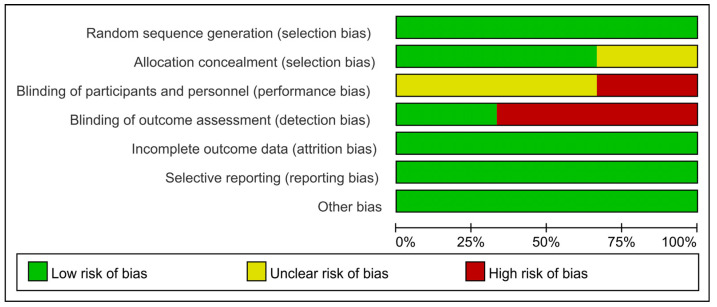
Risk of bias graph. The grading for each domain is based on the Cochrane Risk of Bias tool, with studies classified as low, unclear, or high risk for each domain. The domains assessed include selection bias, performance bias, detection bias, attrition bias, reporting bias and other bias.

**Figure 3:**
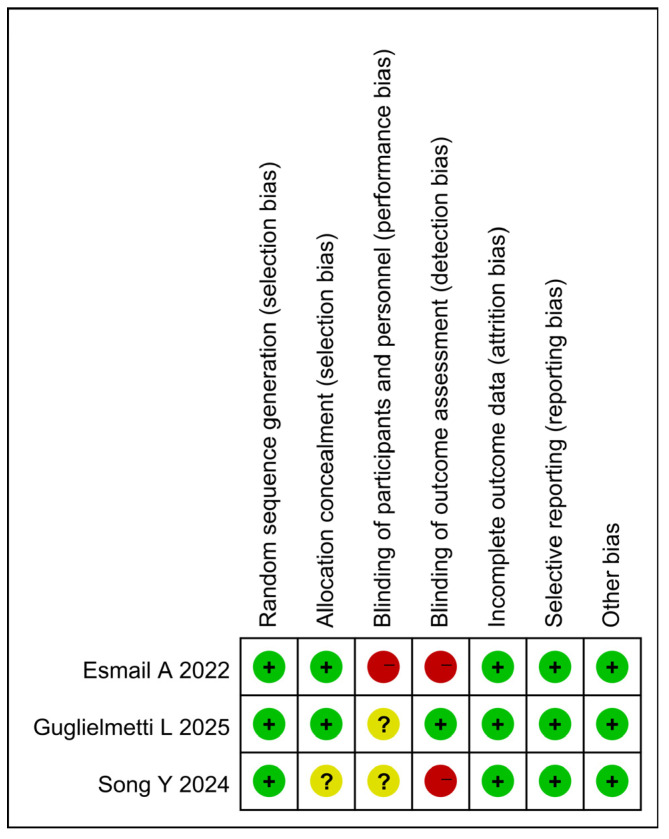
Risk of bias summary. +, Low risk of bias; ?, Unclear risk of bias; −, High risk of bias. Guglielmetti, L [24]; Song, Y [28]; Esmail A [27].

RCTs were assessed using the Cochrane Risk of Bias Assessment Tool. All RCTs showed high or uncleared risks of bias for blinding of performance bias, detection bias (Figure 2 and Figure 3). Observational studies used the NOS. Outcomes ranged from 5 to 9, with a mean score of 7.5; 3 was rated as moderate quality and 6 as high quality (Table 2). The risk of bias for the RCTs centered on the blinding design, with 1 RCT mentioning the infeasibility of the double-blind design due to the different lengths of the regimens and the placebo setup. Given that this may lead to biased results and high heterogeneity, we excluded this non-double-blind RCT from the analysis. Some cohort studies lost points mainly because most of them were single-arm studies with no group comparability; however, the majority of the cohort studies demonstrated good quality, which enhances the reliability of the results.

### 3.4. Meta-Analysis Results

#### 3.4.1. Effectiveness

**Figure 4:**
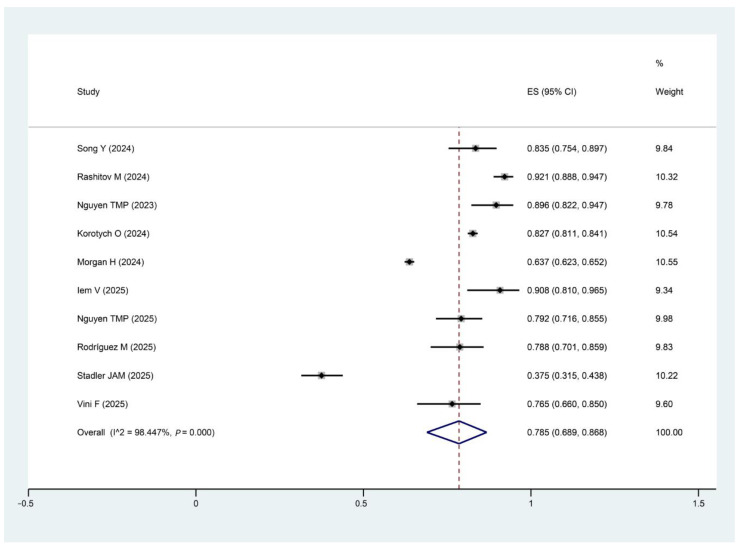
Forest plot of treatment success rate. Forest plot illustrates the effect size (ES) and 95% confidence intervals (CI) of the included studies. Note: The percentage weight for each study is also shown, reflecting the influence of each study on the overall result. The I^2^ statistic (98.45%) and the *p*-value (*p* = 0.00) indicate the level of heterogeneity among the studies. The diamond plot represents the pooled effect size. Song, Y [28]; Rashitov, M [16]; Nguyen, T.M.P [15]; Korotych, O [13]; Morgan, H [29]; Iem, V [30];Nguyen, T.M.P [31]; Rodríguez, M [17]; Stadler, J.A.M [32]; Vini F [33].

10 of the included studies reported treatment success rates, with a pooled treatment success rate of 78.5% (95% CI: 0.69~0.87, I^2^: 98.45%; *p* = 0.00) for the modified shorter regimens, with a total of 8123 patients included (Figure 4). I^2^ statistics indicated substantial between-study heterogeneity.

#### 3.4.2. Safety

For the safety assessment, data on serious adverse events, corrected QT interval (QTc) prolongation, hepatotoxicity, peripheral neuropathy, gastrointestinal symptoms, and optic neuritis were extracted from the included studies and analyzed. Given the variability in definitions and reporting across studies, a narrative synthesis was performed to summarize and present the findings from these safety outcomes (Table 3). Safety outcomes were reported in 6 of the included studies. Overall, 10.0% of patients being treated with modified shorter regimens experienced serious adverse events.

QTc prolongation was reported in 1.8% of patients, though the incidence varied across studies. Hepatotoxicity was observed in 2.6% of patients, while peripheral neuropathy occurred in 0.9%. Gastrointestinal symptoms were present in 1.8% of patients. The incidence of optic neuritis remained low, affecting only 0.3% of patients.

### 3.5. Sensitivity Analysis

A sensitivity analysis was conducted to assess the impact of each individual study on the overall results, with one study removed at a time. The analysis showed that none of the individual studies had a significant influence on the pooled results within the 95% confidence intervals. Therefore, the results of this meta-analysis were found to be relatively reliable. (Supplementary File S3).

### 3.6. Subgroup Analysis

A subgroup analysis was considered to explore potential sources of heterogeneity based on HIV status, treatment regimen and length.

#### 3.6.1. HIV Status

To explore potential sources of heterogeneity, subgroup analysis was performed stratified by HIV status (high, low, and N/A).

**Figure 5:**
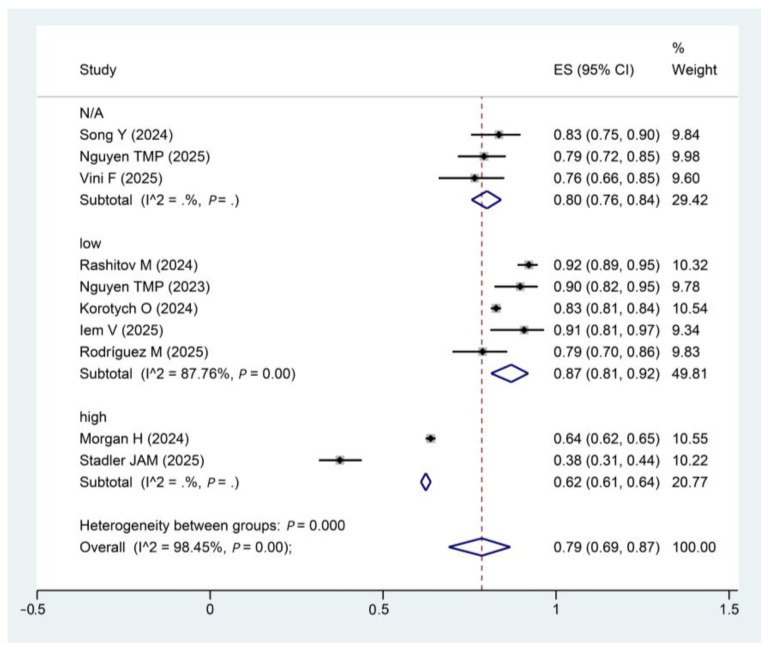
Forest plot of treatment success rates by HIV status subgroup. Note: High, HIV > 50%; low, HIV < 50%; N/A, not available. The diamond plot represents the pooled effect size. Song, Y [28]; Nguyen, T.M.P [15]; Vini F [33]; Rashitov, M [16]; Nguyen, T.M.P [31]; Korotych, O [13]; Morgan, H [29]; Iem, V [30]; Rodríguez, M [17]; Stadler, J.A.M [32].

The subgroup analysis revealed a clear gradient in pooled outcome rates based on HIV status: the rate was highest in the low HIV status subgroup (87%), intermediate in the N/A subgroup (80%), and lowest in the high HIV status subgroup (62%). Heterogeneity between groups (*p* = 0.00) suggests that HIV status is a key factor contributing to the variability observed in the overall results (Figure 5).

#### 3.6.2. Treatment Regimen

**Figure 6:**
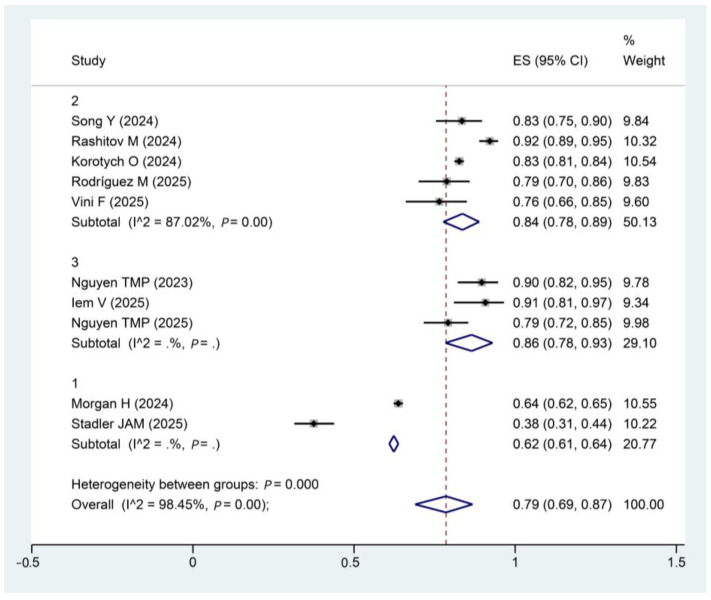
Forest plot of treatment success rates by treatment regimen. Note: Subgroup 1, Bdq + Lfx/Mfx + Cfz + Lzd + E + hINH + Z; Subgroup 2, Bdq + Lzd + Lfx + Cs + Cfz; Subgroup 3, Bdq + Lzd + Lfx + Cfz + Z. The diamond plot represents the pooled effect size. Song, Y [28]; Rashitov, M [16]; Nguyen, T.M.P [15]; Korotych, O [13]; Morgan, H [29]; Iem, V [30]; Nguyen, T.M.P [31]; Rodríguez, M [17]; Vini F [33]; Stadler, J.A.M [32].

To explore potential sources of heterogeneity, we stratified the analysis by treatment regimen (Figure 6). Distinct patterns in pooled treatment success rates were observed across the subgroups. The significant heterogeneity between groups (*p* = 0.000) indicates that the treatment regimen is a key contributor to the variability in the overall results, with the Bdq + Lzd + Lfx + Cs + Cfz (84%) and Bdq + Lzd + Lfx + Cfz + Z (86%) regimens showing higher success rates compared to the Bdq + Lfx/Mfx + Cfz + Lzd + E+hINH + Z (62%) regimen.

#### 3.6.3. Treatment Length

To explore potential sources of heterogeneity, subgroup analysis was conducted based on treatment duration (9 months and over 9 months).

**Figure 7:**
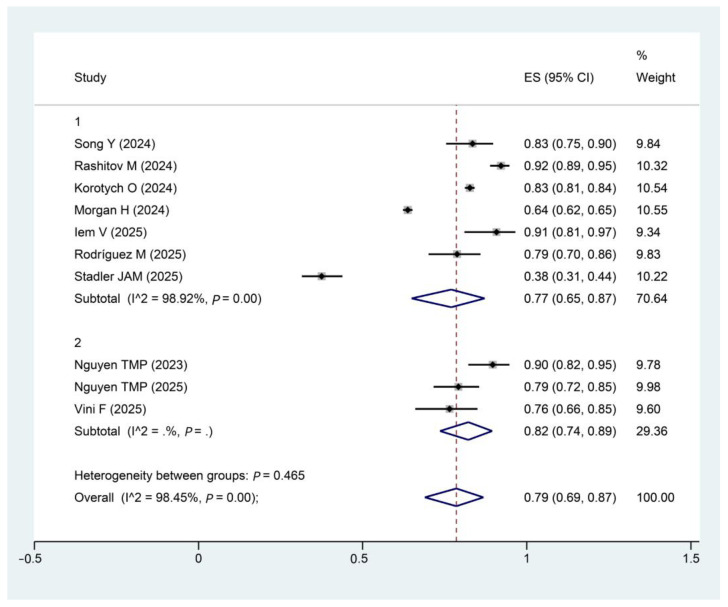
Forest plot of treatment success rates by treatment duration. Note: Subgroup 1, 9 months; Subgroup 2, Over 9 months. The diamond plot represents the pooled effect size. Song, Y [28]; Rashitov, M [16]; Nguyen, T.M.P [15]; Korotych, O [13]; Morgan, H [29]; Iem, V [30]; Nguyen, T.M.P [31];Rodríguez, M [17]; Vini F [33];Stadler, J.A.M [32].

To explore potential sources of heterogeneity, we stratified the analysis by treatment duration. Distinct patterns in pooled treatment success rates were observed across the subgroups. The non-significant heterogeneity between groups (*p* = 0.465) indicates that treatment duration is not the main contributor to overall heterogeneity (Figure 7).

### 3.7. GRADE Certainty of Evidence

The certainty of evidence for the primary outcome (treatment success rate) was evaluated using the GRADE approach. The pooled treatment success rate was 78.5% (95% CI: 68.9% to 86.8%). However, the overall certainty of the evidence was rated as “Very Low”. This rating was primarily due to the observational nature of the included single-arm studies (risk of bias) and the significant unexplained heterogeneity (I^2^ = 98.45%) observed in the analysis (Table 4).

## 4. Discussion

This single-arm meta-analysis was designed to assess the effectiveness and safety of modified shorter regimens containing bedaquiline for the treatment of MDR/RR-TB patients, and a total of 11 studies were included, with 8166 patients. To date, this is the first meta-analysis to be conducted on modified shorter regimens. The pooled treatment success rate of 78.5% indicates the potential effectiveness of the modified shorter regimen for treating MDR/RR-TB. However, this estimate must be interpreted with caution due to the high heterogeneity (I^2^ = 98.45%) and very low certainty of evidence as assessed by the GRADE approach. Given that the majority of the included studies were single-arm observational studies, there is a significant risk of bias, and the variability in the results must be considered. As a result, while the treatment success rate appears promising, the findings should not be over-interpreted. Further, randomized controlled trials are needed to provide higher-quality evidence and confirm the effectiveness of these regimens. Thus, the conclusions drawn from this meta-analysis are tentative and should be viewed in the context of the limitations of the existing data. We also recognized that 1 study, with 4224 participants (57% of the total sample), may have influenced the pooled analysis. However, a sensitivity analysis excluding this study revealed minimal changes in the pooled effect size. The small change in effect size and the overlap of confidence intervals indicate that the study’s impact on the overall results is minimal. Additionally, the incidence of serious adverse events was 10.0% in the included studies reporting safety outcomes, and the most common adverse events were hepatotoxicity (2.6%), gastrointestinal symptoms (1.8%), peripheral neuropathy (0.9%), QTc prolongation (1.8%), and optic neuritis (0.3%). Furthermore, the certainty of evidence is low, attributed to high heterogeneity across studies, potential bias, and variability in outcome reporting. Despite these limitations, the observed outcomes provide preliminary insights into the performance of modified shorter regimens in the included populations, offering reference value for clinical decision-making when standardized regimens are unavailable or inappropriate for individual patients.

QTc prolongation is the most common adverse event associated with antituberculosis drugs in different populations receiving bedaquiline-containing regimens due to potential cardiotoxicity. It is known that the absolute corrected QT measured at 500 ms is thought to increase the risk of potentially fatal arrhythmias [34,35]. Therefore, this adverse event has often been of concern in the design of new therapeutic regimens [36]. In addition to bedaquiline, it is generally considered to be associated with delamanid, fluoroquinolone, and clofazimine [37,38,39], which are included in modified regimens. Although none of the treatment regimens in this study involved delamanid because of the restricted number of included studies, QTc prolongation was reported in 1.8% of patients, but variability in reporting standards and missing data limit conclusions about the regimen’s impact on this adverse event. Concurrent use of QT-prolonging drugs may contribute to this outcome, but the single-arm design prevents definitive attribution. A meta-analysis stating that the combination of bedaquiline and delamanid is safe and tolerable when treating MDR-TB further supports the rationale for the modified regimen design. It has been shown that the combination of bedaquiline with fluoroquinolone and/or clofazimine resulted in an increased incidence of QTc prolongation compared to bedaquiline alone [40], and the modified regimen may be beneficial in overcoming the incidence of QTc prolongation due to the combination of the drugs. However, more reliable clinical studies are needed to confirm this further.

Based on the WHO-recommended shorter regimen of 9–12 months containing bedaquiline, in our study intervention, some original drugs were either replaced or partially replaced as second-line antituberculosis drugs. WHO also recommended that if there is high or confirmed resistance to ethionamide, ethambutol, pyrazinamide, clofazimine, and high-dose isoniazid, then adjustments to the treatment regimen may be made by prioritizing the use of second-line oral medicines [4]. Accordingly, the modified shorter regimen may provide more flexibility for individuals who need an alternative regimen, making the regimen more widely used and applicable, and such novel, more flexible, shorter, less toxic but highly effective regimens to treat drug-resistant TB patients are exactly what is needed in the clinic [41]. In addition, it has been noted that an all-oral second-line regimen based on bedaquiline has shown similar therapeutic microbiological responses to first-line regimens in the drug-resistant TB treatment [42], suggesting the potential therapeutic effect of second-line antituberculosis drug-based regimens. Nevertheless, further investigation is required in the form of large-scale trials to verify these findings.

In summary, this meta-analysis has several strengths. First, modified shorter MDR-TB regimens created in accordance with the hierarchy of TB medications are not well supported by data. This study draws upon the hierarchy of TB medicines, and it is the first meta-analysis to evaluate the effectiveness and safety of modified shorter bedaquiline-containing regimens, which provides evidence in support. Second, we included a total of 8166 multidrug/rifampicin-resistant TB patients with specific data and did not limit the patients’ nationality or age, making the results more generalizable. Moreover, the WHO-recommended shorter regimen had more stringent inclusion criteria and a fixed drug composition, resulting in less applicability. In contrast, modified shorter regimens may provide a more flexible and individualized alternative.

Nevertheless, some limitations should be noted. First, there is no doubt that there is a great deal of heterogeneity among the included studies in this study, which creates uncertainty in our results. The observed heterogeneity in this study may stem from several factors, including variability in diversity in patient populations, non-uniformity of intervention regimens, and differences in treatment lengths. These factors contribute to high heterogeneity and affect the reliability of the pooled results. Through subgroup analysis, we found that HIV status and treatment regimen may be significant sources of heterogeneity. Although subgroup analyses were conducted based on HIV status, treatment regimen, and duration, heterogeneity remained high. This can be attributed to several factors: variability in study design and quality, heterogeneity in patient populations, and inconsistencies in outcome reporting. Despite the limited number of studies and their lack of standardization, we believe the research still holds value, which justifies their inclusion. As a single-arm meta-analysis, the inherent limitations of this design further amplify heterogeneity. The absence of a unified control group in single-arm studies means that treatment effects are typically assessed through pre-post comparisons or indirect external comparisons, which introduces baseline differences that contribute to outcome variability. These baseline differences, such as patient demographics, comorbidities, and prior treatments, are difficult to control for in the absence of a comparator group, leading to susceptibility to confounding. Without a direct comparison to a control group, it is challenging to isolate the effects of the treatment from other factors that may influence outcomes. As a result, the findings of this study should be interpreted with caution, and the conclusions are inherently limited by the lack of comparative data. Additionally, the inclusion of various study types increases the complexity and variability of the pooled results.

Second, the clinical credibility of the modified shorter regimen is questionable due to the paucity of evidence, such as the limited availability of clinical trials in this field, the lack of standardized outcome measures, and the paucity of evidence for specific subgroups; we ultimately included only 2 RCTs, with the remaining studies being observational. The absence of a sufficient number of RCTs fails to allow for meaningful subgroup analysis based on study design. The limitations of the included studies may prevent us from ensuring the uniformity of study quality, which could affect the accuracy and reliability of the pooled results. Further verification is necessary to substantiate its clinical role, and this should be achieved through the implementation of more representative clinical trials.

Third, the number of different treatment regimens and included studies was limited, and we were only able to evaluate the most common adverse events. There may be other potential, rare, and equally important adverse events that were not identified. Furthermore, confounding factors such as the HIV status and prior use of second-line antituberculosis drugs are challenging to control due to the limited number of studies. This highlights the need to interpret the results regarding effectiveness and safety with caution.

## 5. Conclusions

In this single-arm meta-analysis, we found that bedaquiline-containing modified shorter regimens may offer a potentially viable treatment option for MDR/RR-TB patients, which provides an alternative for MDR/RR-TB patients who are unable to opt for a standard regimen due to limited choice of antituberculosis medications and personal factors. Considering the limited strength of the available evidence, further evaluation is required to determine the effectiveness and safety of the modified shorter regimen in larger-scale clinical trials, but single-arm studies with limited resources and small samples are still considered to provide valuable reference evidence.

## Figures and Tables

**Table 1:** Studies Included in the Meta-Analysis.

Author	Year	Country	Study Design	No. of Patients	Males, n (%)	Mean Age (Mean ± SD, Years)	TB Disease	HIV Infected, n (%)	Treatment Regimen	Duration of Treatment	Outcomes		
											Treatment Success, n (%)	Failure, n (%)	Death, n (%)
Guglielmetti L [24]	2025	Georgia, India, Kazakhstan, Lesotho, Pakistan, Peru, South Africa	RCT	115	78 (67.8)	N/A	RR	14 (12.2)	Bdq + Lzd + Lfx + Cfz + Z	9	N/A	N/A	1 (0.9)
Iem V [30]	2025	Lao People’s Democratic Republic	RPCC	65	42 (64.6)	47 ±14	MDR/RR	1 (1.5)	Bdq + Lzd + Lfx + Cfz + Z	9	59 (90.8)	N/A	5 (7.5)
Nguyen TMP [31]	2025	Vietnam	RC	144	111 (77.1)	N/A	RR	N/A	Bdq + Lzd + Lfx + Cfz + Z	9–11	114 (79.2)	2 (1.4)	12 (8.3)
Rodríguez M [17]	2025	Dominican Republic	PC	113	80 (71)	40 ± 15	MDR/RR	14 (12)	Bdq + Lzd + Lfx + Cs + Cfz	9	89 (79)	1 (1)	7 (6)
Fardhdiani V [33]	2025	Ukraine	PC	85	N/A	N/A	MDR	N/A	Bdq + Lzd + Lfx + Cs + Cfz	9–12	65 (76.5)	4 (5)	13 (15)
Stadler JAM [32]	2025	South Africa	PC	248	146 (58.9)	N/A	RR	173 (69.8)	Bdq + Lzd + Mfx + Cfz + E+hINH + Z	9	93 (37.5)	87 (35.1)	20 (8.1)
Rashitov M [16]	2024	Kazakhstan	PC	399	236 (59.1)	N/A	MDR/RR	8 (2)	Bdq + Lzd + Lfx + Cs + Cfz	9	328 (92.1)	17 (4.8)	6 (1.7)
Song Y [28]	2024	China	RCT	115	67 (58.3)	37.1 ± 12.6	MDR	N/A	Bdq + Lzd + Lfx + Cs + Cfz	9	96 (83.5)	N/A	0 (0)
Morgan H [29]	2024	South Africa	RC	4244	2536 (60)	38 ± 13	RR	2826 (67)	Bdq + Lzd + Lfx/Mfx + Cfz + E+hINH + Z	9	2705(64)	47 (1)	853 (20)
Korotych O [13]	2024	13 countries *	PC	2532	1899 (75)	43 ± 2.7	RR	259 (10.2)	Bdq + Lzd + Lfx + Cs + Cfz	9	2093 (82.7)	184 (7.3)	110 (4.3)
Nguyen TMP [15]	2023	Vietnam	PC	106	75 (70.8)	41.2 ± 5.6	RR	1 (0.9)	Bdq + Lzd + Lfx + Cfz + Z	9–11	95 (89.6)	N/A	1 (0.9)

SD, standard deviation; TB, tuberculosis; PC, prospective cohort; RPCC, retrospective and prospective comparative cohort study; N/A, not available; MDR, multidrug-resistant; RR, rifampin-resistant; Bdq, bedaquiline; Lzd, linezolid; Lfx, levofloxacin; Cs, cycloserine; Cfz, clofazimine; RCT, randomized controlled trial; RC, retrospective cohort; Mfx, moxifloxacin; E, ethambutol; hINH, high-dose isoniazid; Z, pyrazinamide; Trd, terizidone; Eto, ethionamide. *, Armenia, Azerbaijan, Belarus, Georgia, Kazakhstan, Kyrgyzstan, Latvia, Lithuania, Republic of Moldova, Tajikistan, Turkmenistan, Ukraine, Uzbekistan.

**Table 2:** Risk of bias assessment of the included studies using the Newcastle–Ottawa quality assessment scale.

Study	Selection	Comparability	Exposure/Outcome	Total Score
Iem V 2025 [30]	4	2	3	9
Nguyen TMP 2025 [31]	4	2	3	9
Rodríguez M 2025 [17]	4	1	3	8
Fardhdiani V2025 [33]	4	2	2	8
Stadler JAM 2025 [32]	4	1	3	8
Rashitov M 2024 [16]	3	-	2	5
Morgan H 2024 [29]	4	2	3	9
Korotych O 2024 [13]	3	-	3	6
Nguyen TMP 2023 [15]	3	-	3	6

**Table 3:** Adverse Events in the studies included in the meta-analysis.

Author	Year	SAEs, n (%)	QTc Prolongation, n (%)	Hepatotoxicity, n (%)	Peripheral Neuropathy, n (%)	Gastrointestinal Symptoms, n (%)	Optic Neuritis, n (%)
Guglielmetti L [24]	2025	16 (13.1)	4 (3.3)	N/A	5 (4.1)	N/A	1 (0.8)
Iem V [30]	2025	8 (12.3)	37 (56.9)	N/A	N/A	N/A	0
Rashitov M [16]	2024	N/A	2 (0.5)	1 (0.3)	15 (3.8)	N/A	1 (0.3)
Song Y [28]	2024	3 (2.6)	26 (22.6)	2 (1.7)	9 (7.8)	1 (0.9)	1 (0.3)
Morgan H [29]	2024	N/A	16 (0.4)	N/A	13 (0.3)	N/A	12 (0.3)
Nguyen TMP [15]	2023	13 (12.0)	4 (3.7)	13 (12.0)	3 (2.7)	3 (2.7)	1 (0.9)
Overall		40 (10.0)	89 (1.8)	16 (2.6)	45 (0.9)	4 (1.8)	16 (0.3)

SAEs, serious adverse events; QTc, corrected QT; N/A, not available.

**Table 4:** GRADE Summary of Findings: Treatment success.

Outcome	Impact	No of Participants (Studies)	Certainty of the Evidence (GRADE)
Treatment Success Rate	The pooled treatment success rate was 78.5% (95% CI: 68.9% to 86.8%).	8123 (10 studies)	⨁◯◯◯ VERY LOW ^a,b^

Note: GRADE Working Group grades of evidence. High certainty: We are very confident that the true effect lies close to that of the estimate of the effect. Moderate certainty: We are moderately confident in the effect estimate. Low certainty: Our confidence in the effect estimate is limited. Very Low certainty: We have very little confidence in the effect estimate. ^a^, Risk of Bias: Downgraded one level. Serious risk of bias was observed in included studies (confounding and lack of blinding in observational designs). ^b^, Inconsistency: Downgraded to one level. Heterogeneity was high (I^2^ = 98.45%, *p* < 0.001), indicating unexplained variability across studies.

## Data Availability

Data is provided within the manuscript or Supplementary Information Files.

## Supplementary Materials

The following supporting information can be downloaded at: https://www.mdpi.com/article/10.3390/pathogens15020130/s1, File S1: PRISMA 2020 Checklist; File S2: Search strategy for each database; File S3: Sensitivity analysis.

## Abbreviations

The following abbreviations are used in this manuscript:

TB	Tuberculosis
MDR-TB	Multidrug-resistant tuberculosis
RR-TB	Rifampicin-resistant tuberculosis
WHO	World Health Organization
MDR/RR-TB	Multidrug-resistant/rifampicin-resistant tuberculosis
RCTs	Randomized controlled trials
NOS	Newcastle–Ottawa Scale
HIV	Human immunodeficiency virus
CIs	Confidence Intervals
GRADE	Grading of Recommendations Assessment, Development, and Evaluation
QTc	Corrected QT interval
