# Supplementary material for: Effectiveness and Safety of Bedaquiline-Containing Modified Shorter Regimens for Multidrug- or Rifampicin-Resistant Tuberculosis: A Single-Arm Meta-Analysis
Source: Pathogens. 2026 Jan 25;15(2):130. doi: 10.3390/pathogens15020130 (PMC12943216; doi:10.3390/pathogens15020130)
Supplement: Supplementary file 1 [file pathogens-15-00130-s001.zip › File S2 Search strategy for each database.pdf]

Pubmed
((("tuberculosis, multidrug resistant"[MeSH Terms] OR "multidrug resistant tuberculosis"[Title/Abstract] OR "tuberculosis multidrug resistant"[Title/Abstract] OR "tuberculosis mdr"[Title/Abstract] OR "mdr tuberculosis"[Title/Abstract] OR "tuberculosis multi drug resistant"[Title/Abstract] OR "multi drug resistant tuberculosis"[Title/Abstract] OR "tuberculosis multi drug resistant"[Title/Abstract] OR "tuberculosis drug resistant"[Title/Abstract] OR "drug resistant tuberculosis"[Title/Abstract] OR "tuberculosis drug resistant"[Title/Abstract]) OR (((rifampicin-resistant tuberculosis[Title/Abstract]) OR (RR-TB[Title/Abstract])) OR (RR-tuberculosis[Title/Abstract]))) AND ((((((((Bedaquiline[Title/Abstract]) OR (Bdq[Title/Abstract])) OR ((levofloxacin[Title/Abstract]) OR (Lfx[Title/Abstract]))) OR ((moxifloxacin[Title/Abstract]) OR (Mfx[Title/Abstract]))) OR ((clofazimine[Title/Abstract]) OR (Cfz[Title/Abstract]))) OR ((cycloserine[Title/Abstract]) OR (Cs[Title/Abstract]))) OR ((linezolid[Title/Abstract]) OR (Lzd[Title/Abstract]))) OR ((delamanid[Title/Abstract]) OR (Dlm[Title/Abstract]))) AND ((((short treatment regimen) OR (modified short treatment regimen)) OR (Shortened treatment regimens)) OR (modified Shortened treatment regimens)))) AND ((("randomized controlled trial"[Publication Type] OR "randomized"[Title/Abstract] OR "placebo"[Title/Abstract])) OR ((((((((("Case-Control Studies"[Mesh]) OR ("Cohort Studies"[Mesh])) OR (Cohort Study[Title/Abstract])) OR (Studies, Cohort[Title/Abstract])) OR (Study, Cohort[Title/Abstract])) OR (Studies, Concurrent[Title/Abstract])) OR (Concurrent Study[Title/Abstract])) OR (Study, Concurrent[Title/Abstract])) OR (Concurrent Studies[Title/Abstract])))

Embase	
#1	'multidrug resistant tuberculosis'/exp OR 'multidrug resistant tuberculosis':ab,ti OR 'tuberculosis multidrug resistant':ab,ti OR ' tuberculosis mdr':ab,ti OR 'mdr tuberculosis ':ab,ti OR'tuberculosis multi drug resistant':ab,ti OR 'multi drug resistant tuberculosis':ab,ti OR 'tuberculosis multi drug resistant':ab,ti OR 'tuberculosis drug resistant':ab,ti OR'drug resistant tuberculosis':ab,ti OR 'tuberculosis drug resistant':ab,ti 'rifampicin-resistant tuberculosis':ab,ti OR 'RR-TB':ab,ti OR 'RR-tuberculosis':ab,ti
#2	'Bedaquiline':ab,ti OR 'Bdq':ab,ti OR 'levofloxacin':ab,ti OR'Lfx ':ab,ti OR 'moxifloxacin':ab,ti 'Mfx':ab,ti OR 'clofazimine':ab,ti OR 'Cfz':ab,ti OR 'cycloserine':ab,ti OR 'Cs':ab,ti OR 'linezolid':ab,ti OR 'Lzd ':ab,ti OR 'delamanid':ab,ti 'Dlm':ab,ti
#3	'short treatment regimen':ab,ti OR 'modified short treatment regimen ':ab,ti OR 'Shortened treatment regimens':ab,ti OR'modified Shortened treatment regimens':ab,ti OR 'shorter regimen ':ab,ti OR 'Short regimens':ab,ti

Web of science	
#1	TS=(tuberculosis, multidrug resistant OR multidrug resistant tuberculosis OR tuberculosis multidrug resistant OR tuberculosis mdr OR mdr tuberculosis OR tuberculosis multi drug resistant OR multi drug resistant tuberculosis OR tuberculosis multi drug resistant OR tuberculosis drug resistant OR drug resistant tuberculosis OR tuberculosis drug resistant OR rifampicin resistant tuberculosis OR RR-TB OR RR-tuberculosis)
#2	TS=(randomized controlled trial OR randomized OR placebo OR Case-Control Studies OR Cohort Studies OR cohort study OR studies cohort OR study cohort OR studies concurrent OR concurrent study OR study concurrent OR concurrent studies)
#3	TS=(Bedaquiline OR Bdq OR levofloxacin OR Lfx OR moxifloxacin OR Mfx OR clofazimine OR Cfz OR cycloserine OR Cs OR linezolid OR Lzd OR delamanid OR Dlm)
#4	TS=(shorter regimen* OR short treatment regimen* OR modified short treatment regimen* OR Shortened treatment regimen* OR modified Shortened treatment regimen* OR mSTR)
#5	#3 and #4
#6	#1 and #2 and #5

Cochrane library	
#1	MeSH descriptor: [Tuberculosis, Multidrug-Resistant] explode all trees
#2	(multidrug resistant tuberculosis)ti,ab,kw OR(tuberculosis multidrug resistant):ti,ab,kw OR(tuberculosis mdr):ti,ab,kw OR(mdr tuberculosis):ti,ab,kw OR (tuberculosis multi drug resistant):ti,ab,kw
#3	(multi drug resistant tuberculosis)ti,ab,kw OR(tuberculosis multi drug resistant)ti,ab,kw OR(tuberculosis mdr)ti,ab,kw OR(tuberculosis drug resistant)ti,ab,kw OR(drug resistant tuberculosis)ti,ab,kw
#4	(tuberculosis drug resistant):ti,ab,kw OR (rifampicin-resistant tuberculosis):ti,ab,kw OR (RR-TB):ti,ab,kw OR(RR-tuberculosis):ti,ab,kw
#5	(Bedaquiline):ti,ab,kw OR(Bdq)ti,ab,kw OR(levofloxacin)ti,ab,kw OR(Lfx):ti,ab,kw AND(moxifloxacin)ti,ab,kw
#6	(Mfx):ti,ab,kw OR(clofazimine)t,ab,kw OR(Cfz):ti,ab,kw OR(cycloserine):ti,ab,kw AND(Cs):ti,ab,kw
#7	(linezolid):ti,ab,kw OR(Lzd):ti,ab,kw OR(delamanid):ti,ab,kw OR(DIm):ti,ab,kw
#8	(short treatment regimen):ti,ab,kw OR(short treatment regimens):ti,ab,kw OR(modified short treatment regimen):ti,ab,kw OR(modified short treatment regimens)ti,ab,kw AND(shorter regimen)ti,ab,kw
#9	(short regimens)ti,ab,kw
#10	#1 or #2 or #3 or #4
#11	#5 or #6 or #7
#12	#8 or #9
#13	#10 or #11
#14	#12 and #13
