# Supplementary figures and images for: Effectiveness and Safety of Bedaquiline-Containing Modified Shorter Regimens for Multidrug- or Rifampicin-Resistant Tuberculosis: A Single-Arm Meta-Analysis
Source: Pathogens. 2026 Jan 25;15(2):130. doi: 10.3390/pathogens15020130 (PMC12943216; doi:10.3390/pathogens15020130)

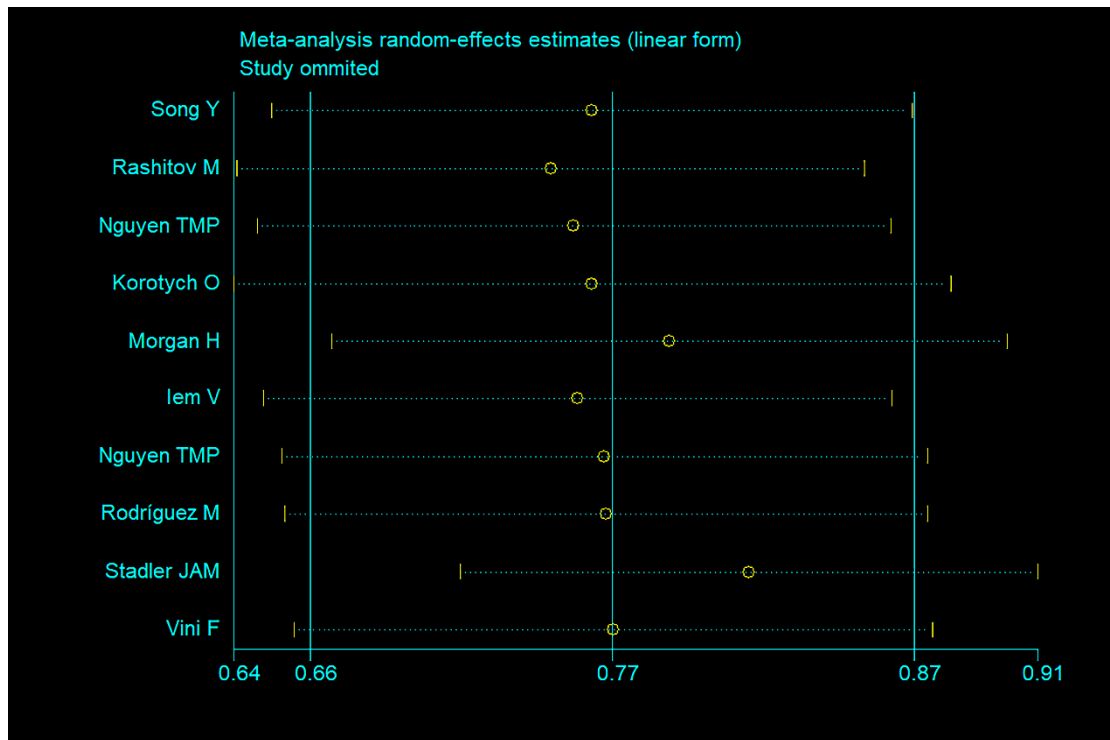

Figure S1. Sensitivity analysis

Supplement: Supplementary file 1 [file pathogens-15-00130-s001.zip › File S3 Sensitivity analysis.pdf]
